# Bayesian differential analysis of gene regulatory networks exploiting genetic perturbations

**DOI:** 10.1186/s12859-019-3314-3

**Published:** 2020-01-09

**Authors:** Yan Li, Dayou Liu, Tengfei Li, Yungang Zhu

**Affiliations:** 1 College of Computer Science and Technology, Jilin University, Changchun, 130012 China; 2 Key Laboratory of Symbolic Computation and Knowledge Engineering of Ministry of Education, Jilin University, Changchun, 130012 China

**Keywords:** Gene regulatory networks, Gene expression, Genetic perturbations, Structural equation models, Differential GRN, Bayesian inference

## Abstract

**Background:**

Gene regulatory networks (GRNs) can be inferred from both gene expression data and genetic perturbations. Under different conditions, the gene data of the same gene set may be different from each other, which results in different GRNs. Detecting structural difference between GRNs under different conditions is of great significance for understanding gene functions and biological mechanisms.

**Results:**

In this paper, we propose a Bayesian Fused algorithm to jointly infer differential structures of GRNs under two different conditions. The algorithm is developed for GRNs modeled with structural equation models (SEMs), which makes it possible to incorporate genetic perturbations into models to improve the inference accuracy, so we name it BFDSEM. Different from the naive approaches that separately infer pair-wise GRNs and identify the difference from the inferred GRNs, we first re-parameterize the two SEMs to form an integrated model that takes full advantage of the two groups of gene data, and then solve the re-parameterized model by developing a novel Bayesian fused prior following the criterion that separate GRNs and differential GRN are both sparse.

**Conclusions:**

Computer simulations are run on synthetic data to compare BFDSEM to two state-of-the-art joint inference algorithms: FSSEM and ReDNet. The results demonstrate that the performance of BFDSEM is comparable to FSSEM, and is generally better than ReDNet. The BFDSEM algorithm is also applied to a real data set of lung cancer and adjacent normal tissues, the yielded normal GRN and differential GRN are consistent with the reported results in previous literatures. An open-source program implementing BFDSEM is freely available in Additional file 1.

## Background

GRNs visually reflect the gene-gene interactions, which are significant for understanding gene functions and biological activities. In the past few years, a series of inference algorithms have been proposed to reconstruct topology structures of GRNs. Some computational methods were only developed to infer GRNs from gene expression data, such as Boolean networks [1], mutual information models [2, 3], Gaussian Graphical models [4, 5], Bayesian networks [6, 7] and linear regression models [8, 9]; several other methods were also built to improve the accuracy of inference by integrating genetic perturbations with gene expression data, among which the algorithms based on SEMs [10–13] are one of the most popular approaches.

Most of the existing computational methods mainly focus on inferring GRNs under one single condition, but can not jointly identify changes in GRN structures when the condition (e.g. environments, tissues, diseases) changes. However, the differential analysis of GRNs under different conditions is also of significant importance to identify gene functions, discover biological mechanisms of different tissues and find genes related to diseases [14–16].

Intuitively, a naive approach for identifying the structure difference between GRNs under different conditions is to separately infer GRNs with existing methods and identify the difference by comparing the resulted GRNs. However, in this way, the similarity between GRNs are not taken into consideration, so the accuracy is probably unsatisfactory. Recently, several algorithms were developed to jointly infer GRNs from gene expression data under different conditions. For example, Mohan et al. [17] and Danaher et al. [18] proposed penalized algorithms based on multiple Gaussian graphical models to jointly infer GRNs under different conditions exploiting the similarities and differences between them. Wang et al. [19] developed an efficient proximal gradient algorithm to jointly infer GRNs modeled with linear regression models and identify the changes in the structure. However, the Gaussian graphical models can not identify directed networks, and the above algorithms were all developed for inferring GRNs from a single data source. Zhou and Cai [20] modeled GRNs with SEMs to integrate genetic perturbations with gene expression data, and developed a fused sparse SEM (FSSEM) algorithm to make joint inference. Ren and Zhang [21] proposed a re-parametrization based differential analysis algorithm for SEMs (ReDNet), they re-parameterized the pair-wise SEMs as one integrated SEM incorporating the averaged GRN and differential GRN, and then identified the difference GRN directly from the integrated model. Both FSSEM and ReDNet made joint differential analysis for directed GRNs modeled with SEMs, their simulation studies demonstrated that FSSEM and ReDNet significantly outperformed naive approaches based on SML [13] and 2SPLS [22], respectively.

In this paper, we propose a Bayesian Fused Differential analysis algorithm for GRNs modeled with SEMs (BFDSEM) to jointly infer pair-wise GRNs under different conditions. Following the fact that GRNs under different conditions differ slightly from each other, the sparsity of separate GRNs and differential GRN are both taken into consideration. In addition, there is no limitation on the structure of GRNs, that is, both directed acyclic GRNs (DAGs) and directed cyclic GRNs (DCGs) are supported. Computer simulations are run to compare the performance of our proposed BFDSEM to FSSEM and ReDNet, the results demonstrate that BFDSEM has somewhat consistent results with FSSEM and has better performance than ReDNet.

## Preliminaries

### The Bayesian Fused Lasso for linear regression models

Linear regression models can be represented as follows:

1 $$\begin{array}{@{}rcl@{}}  \mathbf{y} = \mathbf{X}\boldsymbol{\beta} + \mathbf{e}. \end{array} $$

where **X=[x**_1_,**x**_2_,⋯,**x**_*p*_] is the design matrix including *p* predictor variables, **y**=[*y*_1_,*y*_2_,⋯,*y*_*n*_]^*T*^ denotes the response vector and ***β***=[*β*_1_,*β*_2_,⋯,*β*_*p*_]^*T*^ is the coefficient vector to be estimated.

Tibshirani [28] proposed Lasso with *l*_1_ penalty on parameters to realize variable selection and parameter estimation simultaneously, the Lasso estimator of Eq. (1) is given by

2 $$\begin{array}{@{}rcl@{}}  \widehat{\boldsymbol{\beta}}^{\textup{L}}=\textup{arg min}_{\boldsymbol{\beta}} \left\{\left\| \mathbf{y} - \mathbf{X}\boldsymbol{\beta} \right\|_{2}^{2}+\lambda \sum_{j=1}^{p}| \beta_{j} | \right\}. \end{array} $$

In a Bayesian framework, the Lasso can be interpreted as the Bayesian posterior mode under independent Laplace priors [28, 29]. As suggested by Park and Casella in [29], the conditional Laplace prior of ***β*** can be represented as a scale mixture of normals with an exponential mixing density

3 $$ {}\begin{aligned}  \pi \left(\boldsymbol{\beta} |\sigma^{2} \right) &= \prod_{j=1}^{p}\frac{\lambda }{2\sqrt{\sigma^{2}}} \textup{exp}\left\{\frac{-\lambda | \beta_{j} |}{\sqrt{\sigma^{2}}}\right\} \\ &= \prod_{j=1}^{p} \int_{0}^{\infty }\frac{1}{\sqrt{2\pi\sigma^{2}\tau_{j}^{2}}}\textup{exp}\left\{\frac{-|\beta_{j}|^{2}}{2\sigma^{2}\tau_{j}^{2}}\right\}\psi \textup{exp}\left\{-\psi \tau_{j}^{2}\right\}d\tau_{j}^{2} \end{aligned}  $$

where *σ*^2^ could be assign a noninformative prior or any conjugate Inverse-Gamma prior, and *ψ* is equivalent to the tuning parameter *λ* as in Eq. (2) that controls the degree of sparsity. After integrating out $\tau _{1}^{2},\tau _{2}^{2},\cdots,\tau _{p}^{2}$, the conditional prior on ***β*** has the desired Laplace form [34]. From this relationship, the Bayesian formulation of Lasso as given in [29] is given by the following hierarchical prior.

4 $$ {}\begin{aligned}  \boldsymbol{\beta}|\sigma^{2}, \tau_{1}^{2},\tau_{2}^{2},\cdots,\tau_{p}^{2} &\sim \textup{N}_{p}\left(\mathbf{0},\sigma^{2}\mathbf{D}_{\tau}\right), \mathbf{D}_{\tau}=\textup{diag}\left(\tau_{1}^{2},\tau_{2}^{2},\cdots,\tau_{p}^{2}\right) \\ \tau_{j}^{2}|\psi &\sim \textup{Exp}(\psi), j=1,2,\cdots,p. \end{aligned}  $$

where N_*p*_(***μ***,***Σ***) denotes *p*-variate normal distribution with mean vector ***μ*** and covariance matrix ***Σ***, and Exp(*ψ*) denotes exponential distribution with rate parameter *ψ*.

A series of extensions of Lasso such as SCAD [30], Elastic net [31], fused Lasso [32], adaptive Lasso [33] were developed for various applications. The fused Lasso penalizes both the coefficients and the differences between adjacent coefficients with *l*_1_-norm, the estimator of fused Lasso for Eq. (1) is given by

5 $$ {}\begin{aligned}  \widehat{\boldsymbol{\beta}}^{\textup{FL}}=\textup{arg min}_{\boldsymbol{\beta}} \left\{\left\| \mathbf{y} - \mathbf{X}\boldsymbol{\beta} \right\|_{2}^{2}+ \lambda_{1} \sum_{j=1}^{p}| \beta_{j} | +\lambda_{2} \sum_{j=1}^{p-1}| \beta_{j+1}-\beta_{j} | \right\} \end{aligned}  $$

Kyung et al. proposed the Bayesian interpretation of fused Lasso in [34]. The conditional prior can be expressed as

6 $$ \begin{aligned}  \pi \left(\boldsymbol{\beta}|\sigma^{2} \right) \propto \textup{exp}\left(-\frac{\lambda_{1} }{\sigma}\sum_{j=1}^{p}|\beta_{j}| - \frac{\lambda_{2} }{\sigma}\sum_{k=1}^{p-1}|\beta_{k+1} - \beta_{k}| \right) \end{aligned}  $$

where *λ*_1_ and *λ*_2_ are tuning parameters. They provide the theoretical asymptotic limiting distribution and a degrees of freedom estimator. Following the way of Bayesian Lasso, this prior can be represented as the following hierarchical form:

7 $$ {}\begin{aligned}  \boldsymbol{\beta}|\sigma^{2}, \tau_{1}^{2},\tau_{2}^{2},\cdots,\tau_{p}^{2}, \omega_{1}^{2},\omega_{2}^{2},\cdots,\omega_{p-1}^{2} &\sim \textup{N}_{p}(\mathbf{0},\sigma^{2}\Sigma_{\beta}),\\ \tau_{j}^{2}|\psi_{1} &\sim \textup{Exp}(\psi_{1}), j=1,2,\cdots,p \\ \omega_{k}^{2}|\psi_{2} &\sim \textup{Exp}(\psi_{2}), k=1,2,\cdots,p-1. \end{aligned}  $$

where $\tau _{1}^{2},\tau _{2}^{2},\cdots,\tau _{p}^{2}, \omega _{1}^{2},\omega _{2}^{2},\cdots,\omega _{p-1}^{2}$ are mutually independent, and *Σ*_*β*_ is a tridiagonal matrix with main diagonal=$\left \{\frac {1}{\tau _{j}^{2}}+\frac {1}{\omega _{j-1}^{2}}+\frac {1}{\omega _{j}^{2}},j=1,2,\cdots,p\right \}$ and off diagonal $\left \{-\frac {1}{\omega _{k}^{2}},k=1,2,\cdots,p-1\right \}$, $\frac {1}{\omega _{0}^{2}}$ and $\frac {1}{\omega _{p}^{2}}$ are defined as 0 for convenience.

As suggested by Park and Casella [29], there are two common approaches to estimate the tuning parameters: one is to estimate them through marginal likelihood implemented with an EM/Gibbs algorithm [36]; another way is to assign a Gamma hyperprior on each tuning parameter, and put them into the hierarchical models to estimate it with a Gibbs sampler.

### GRNs modeled with SEMs

As in [10–13], genetic perturbations can be incorporated into SEMs to infer GRNs and result in better performance. The perturbations could be various, such as the expression Quantitative Trait Loci (eQTLs) and the Copy Number Variants (CNVs). In this paper we consider the variations observed on the cis-eQTLs. Suppose we have expression levels of *p* genes and genotypes of *q* cis-eQTLs observed from *n* individuals. Let **Y**=[**y**_1_,**y**_2_,⋯,**y**_*p*_] be an *n*×*p* gene expression matrix, **X=[x**_1_,**x**_2_,⋯,**x**_*q*_] be an *n*×*q* cis-eQTL matrix. Then the GRN can be modeled with the following SEM:

8 $$\begin{array}{@{}rcl@{}}  \mathbf{Y = YB + XF + E}, \end{array} $$

where the *p*×*p* matrix **B** is the adjacency matrix defining the structure of a GRN, *B*_*ij*_ represents the regulatory effect of the *i*th gene on the *j*th gene; and the *q*×*p* matrix **F** is composed of the regulatory effects of cis-eQTLs, in which *F*_*km*_ denotes the effect of the *k*th cis-eQTL on the *m*th gene. It is often assumed that every gene has no effect on itself, which implies *B*_*ii*_=0 for *i* = 1, ⋯,*p*. To ensure the identifiable of GRNs, we assume there is at least one unique cis-eQTL for each gene.

Let **y**_*i*_=[*y*_1*i*_,*y*_2*i*_,⋯,*y*_*ni*_]^*T*^,*i*=1,⋯,*p* be the *i*th column of **Y**, denoting expression levels of the *i*th gene observed from *n* individuals. And let **B**_*i*_,*i*=1,⋯,*p* be the *i*th column of **B**. As mentioned before, the *i*th gene is considered to have no effect on itself, meaning that the *i*th entry of **B**_*i*_ is known to be zero, so this entry can be removed before inference to reduce the computation complexity. Correspondingly, the *i*th column of **Y** needs also to be removed. Then we can split Eq. (8) into *p* SEMs, in which the *i*th SEM as follows describes how much other genes and corresponding cis-eQTLs affect the *i*th gene.

9 $$\begin{array}{@{}rcl@{}}  \mathbf{y}_{i} = \mathbf{Y}_{-i}\mathbf{b}_{i} +\mathbf{Xf}_{i} +\mathbf{e}_{i}, i = 1,\cdots, p, \end{array} $$

where *n*×1 vector **y**_*i*_ is the *i*th column of **Y** and *n*×(*p*−1) matrix **Y**_−*i*_ refers to **Y** excluding the *i*th column; (*p*−1)×1 vector **b**_*i*_ is the *i*th column of **B** excluding the *i*th row; *q*×1 vector **f**_*i*_ denotes the *i*th column of **F**; *n*×1 vector **e**_*i*_ represents the residual error vector, in which all entries are modeled as independent and identical normal distributions with zero mean and variance *σ*^2^.

### GRNs under different conditions

In this paper, we mainly focus on the joint inference of GRNs under different conditions. We denote the expression levels of *p* genes under two different conditions as $\mathbf {Y}^{(k)} = \left [\mathbf {y}^{(k)}_{1}, \mathbf {y}^{(k)}_{2},\cdots, \mathbf {y}^{(k)}_{p}\right ], k=1, 2$. Similarly, the genotypes of cis-eQTLs under two conditions are represented as $\mathbf {X}^{(k)} = \left [\mathbf {x}^{(k)}_{1}, \mathbf {x}^{(k)}_{2},\cdots, \mathbf {x}^{(k)}_{q}\right ], k=1,2$. Based on the SEM introduced in the previous subsection, we can represent two pair-wise GRNs as

10 $$\begin{array}{@{}rcl@{}}  \mathbf{Y}^{(k)} = \mathbf{Y}^{(k)}\mathbf{B}^{(k)} + \mathbf{X}^{(k)}\mathbf{F}^{(k)} + \mathbf{E}^{(k)},\; \; \; k= 1,2, \end{array} $$

and further represent the sub-models as

11 $$\begin{array}{@{}rcl@{}}  \mathbf{y}^{(k)}_{i} = \mathbf{Y}^{(k)}_{-i}\mathbf{b}^{(k)}_{i} +\mathbf{X}^{(k)}\mathbf{f}^{(k)}_{i} +\mathbf{e}^{(k)}_{i},\; \; \; i = 1,\cdots,p,\; k=1,2, \end{array} $$

where **B**^(*k*)^ depict the structures of two GRNs under different conditions, which contain coefficients for the direct causal effects of the genes on each other.

As discussed above, $\mathbf {f}^{(k)}_{i}$ is sparse and the locations of nonzero entries have been obtained via pretreatment. We assume the row index set of nonzero entries of $\mathbf {f}^{(k)}_{i}$ as $S^{(k)}_{i}$, so in the *i*th model of Eq. (11), **X**^(*k*)^ can be reduced to a matrix $\mathbf {X}^{(k)}_{S^{(k)}_{i}}$ that only contains the columns whose indices are in $S^{(k)}_{i}$. Accordingly, $\mathbf {f}^{(k)}_{i,S^{(k)}_{i}}$ is a reduced form of $\mathbf {f}^{(k)}_{i}$ that only contains the rows whose indices are in $S^{(k)}_{i}$.

### The identifiability of SEMs

Our main goal is to infer the adjacency matrices **B**^(1)^ and **B**^(2)^ from SMEs as in Eq. (10), and identify the difference between them (*Δ***B**=**B**^(1)^−**B**^(2)^) in the meanwhile. Without any knowledge about the GRNs, no restriction is imposed on the structures specified by the adjacency matrices, that is to say, GRNs modeled with SEMs are considered as general directed networks that can possibly be DAGs or DCGs.

As mentioned before, we make some standard assumptions that are used by most popular GRN inference algorithms to ensure model identifiability. For example, the error terms $\mathbf {e}^{(k)}_{i}$ are assumed as independent and identical normal distributions, and the diagonal entries of **B**^(*k*)^ are all assumed to be zero so that there is no self-loop in GRNs.

While DAGs are always identified under the above assumptions, the identifiability of DCGs need further studies because of the challenge in model equivalence [11]. To make meaningful inference, it is important to have as small a set of equivalent models as possible [12]. Logsdon et al. [12] investigated this issue for DCGs in detail in their "Recovery" Theorem. According to their discussion, under the assumption that each gene is directly regulated by a unique nonempty set of cis-eQTLs, there will exist multiple equivalent DCGs, and the perturbation topology can completely change among equivalent DCGs. Furthermore, as in the Lemma of the "Recovery" Theorem, if we know which gene each cis-eQTL feeds into, then the cardinality of the equivalence class is reduced to one, that is, a unique DCG can be inferred. Therefore, we make the assumption that the the loci of the *q* cis-eQTLs have been determined by an existing eQTL method in advance, but the size of each regulatory effect is still unknown. In this way, the perturbation topology is determined, and a unique DCG can be the identified.

Now that the identifiability of SEMs are guaranteed for both DAGs and DCGs with appropriate assumptions, the pair-wise GRNs can be inferred by estimating **B**^(1)^ and **B**^(2)^ column by column by solving Eq. (11).

## Methods

### Joint inference model based on SEMs

By defining $\mathbf {W}_{i}^{(k)} = \left [\mathbf {Y}^{(k)}_{-i}, \mathbf {X}^{(k)}_{S^{(k)}_{i}}\right ]$, $\boldsymbol {\beta }^{(k)}_{i} = \left [\mathbf {b}^{(k)}_{i}, \mathbf {f}^{(k)}_{i,S^{(k)}_{i}} \right ]^{T}$, Eq. (11) can be rewritten as a linear type model

12 $$\begin{array}{@{}rcl@{}}  \mathbf{y}^{(k)}_{i}= \mathbf{W}_{i}^{(k)}\boldsymbol{\beta}^{(k)}_{i}+ \mathbf{e}^{(k)}_{i},\; \; \; i = 1,\cdots,p,\;k= 1,2. \end{array} $$

Therefore, we can first solve Eq. (12) by adopting appropriate regularized linear regression method and then extract $\mathbf {b}^{(k)}_{i}$ from $\boldsymbol {\beta }^{(k)}_{i}$.

As is known, a gene is usually regulated by a small number of genes, meaning that most entries in ***β***^(*k*)^ are equal to zero [23–26]. In addition, pair-wise GRNs under different conditions are biologically considered to be similar, that is to say, most entries in *Δ****β***=***β***^(1)^−***β***^(2)^ are also equal to zero [27]. In order to satisfy the sparsity of both the separate GRNs and the differential GRN, we penalize both ***β***^(*k*)^ and *Δ****β*** with *l*_1_-norm, which would yield the following optimization problem [19]:

13 $$ {}\begin{aligned}  (\widehat{\boldsymbol{\beta}}^{(1)}_{i},\widehat{\boldsymbol{\beta}}^{(2)}_{i}) = &\textup{arg min}_{\boldsymbol{\beta}^{(1)}_{i},\boldsymbol{\beta}^{(2)}_{i}} \left\{{\vphantom{\sum_{2}^{3}}}\right. \!\left\| \mathbf{y}^{(1)}_{i} - \mathbf{W}_{i}^{(1)}\boldsymbol{\beta}^{(1)}_{i} \right\|_{2}^{2} \!\\&+\! \left\| \mathbf{y}^{(2)}_{i} - \mathbf{W}_{i}^{(2)}\boldsymbol{\beta}^{(2)}_{i} \right\|_{2}^{2}+ \lambda_{1}\left(\left\| \boldsymbol{\beta}^{(1)}_{i} \right\|_{1} + \left\| \boldsymbol{\beta}^{(2)}_{i} \right\|_{1}\right)\\ &+ \lambda_{2}\left\| \boldsymbol{\beta}^{(1)}_{i} -\boldsymbol{\beta}^{(2)}_{i} \right\|_{1}\left.{\vphantom{\sum_{2}^{3}}}\right\}, \; \; \;i = 1, \cdots, p, \end{aligned}  $$

where the *l*_1_-norms $\lambda _{1}\left (\left \| \boldsymbol {\beta }^{(1)}_{i} \right \|_{1} + \left \| \boldsymbol {\beta }^{(2)}_{i} \right \|_{1}\right)$ and $\lambda _{2}\left \| \boldsymbol {\beta }^{(1)}_{i} -\boldsymbol {\beta }^{(2)}_{i} \right \|_{1}$ are introduced to fulfill the sparsity of corresponding parameters, *λ*_1_>0 and *λ*_2_>0 are tuning parameters used to control the sparsity levels.

Inspired by the optimization model in Eq. (13), we re-parameterize the pair-wise re-parameterized SEMs as in Eq. (12) to an integrated model as follows,

14 $$\begin{array}{@{}rcl@{}}  \mathbf{y}_{i}= \mathbf{W}_{i}\boldsymbol{\beta}_{i}+ \mathbf{e}_{i},\; \; \; i = 1,\cdots,p, \end{array} $$

where $\mathbf {y}_{i}=\mathbf {y}_{i}^{(1)}+\mathbf {y}_{i}^{(2)}$, $\mathbf {W}_{i} = \left [\mathbf {W}_{i}^{(1)},\mathbf {W}_{i}^{(2)}\right ]$, $\boldsymbol {\beta }_{i} = \left [\boldsymbol {\beta }^{(1)}_{i},\boldsymbol {\beta }^{(2)}_{i}\right ]^{T}$ and $\mathbf {e}_{i}=\mathbf {e}_{i}^{(1)}+\mathbf {e}_{i}^{(2)}$. By denoting the dimension of $S_{i}^{(k)}$ as *q*_*i*_, the dimension of $\boldsymbol {\beta }^{(k)}_{i}$ can be easily expressed as *p*_*i*_=*p*−1+*q*_*i*_. Therefore, **y**_*i*_ and **e**_*i*_ are *n*×1 vectors, **W**_*i*_ is an *n*×2*p*_*i*_ design matrix and ***β***_*i*_ is a 2*p*_*i*_×1 vector containing all unknown parameters to be estimated. Then, the optimization problem in Eq. (13) can be transferred to

15 $$ \begin{aligned}  \widehat{\boldsymbol{\beta}}_{i} &= \textup{arg min}_{\boldsymbol{\beta}_{i}} \left\{ \left\| \mathbf{y}_{i} - \mathbf{W}_{i}\boldsymbol{\beta}_{i} \right\|_{2}^{2} + \lambda_{1}\sum_{j=1}^{2p_{i}} |\boldsymbol{\beta}_{i,j}|\right. \\&\quad+\left. \lambda_{2}\sum_{k=1}^{p_{i}} |\boldsymbol{\beta}_{i,p_{i}+k} -\boldsymbol{\beta}_{i,k}|\right\}, \; \; \;i = 1, \cdots, p. \end{aligned}  $$

In the subsequent section, we infer Eq. (15) in a Bayesian framework by developing a novel appropriate prior to fulfill the required sparsity and estimating the parameters with a Gibbs sampler.

### The BFDSEM algorithm

In this section, we develop the BFDSEM algorithm via a novel hierarchical prior for Eq. (14) to solve the optimization problem as in Eq. (15). Referring to the Bayesian fused Lasso [35], the prior for ***β***_***i***_ is defined as

16 $$ {}\begin{aligned}  \pi \left(\boldsymbol{\beta}_{i} |\sigma^{2} \right) &= \prod_{j=1}^{2p_{i}}\frac{\lambda_{1} }{2\sqrt{\sigma^{2}}} \textup{exp}\left\{\frac{-\lambda_{1} | \beta_{i,j} |}{\sqrt{\sigma^{2}} }\right\}\\&\quad\prod_{k=1}^{p_{i}}\frac{\lambda_{2} }{2\sqrt{\sigma^{2}}} \textup{exp}\left\{\frac{-\lambda_{2} | \beta_{i,p_{i}+k} - \beta_{i,k} |}{\sqrt{\sigma^{2}}}\right\}\! \propto \prod_{j=1}^{2p_{i}} \int_{0}^{\infty }\frac{1}{\sqrt{2\pi\sigma^{2}\tau_{j}^{2}}}\\ &\textup{exp}\left\{\frac{-|\beta_{i,j}|^{2}}{2\sigma^{2}\tau_{j}^{2}}\right\}\psi_{1,j} \textup{exp}\left\{-\psi_{1,j} \tau_{j}^{2}\right\}d\tau_{j}^{2} \times \prod_{k=1}^{p_{i}} \int_{0}^{\infty }\frac{1}{\sqrt{2\pi\sigma^{2}\omega_{k}^{2}}}\\ &\textup{exp}\left\{\frac{-|\beta_{i,p_{i}+k}-\beta_{i,k}|^{2}}{2\sigma^{2}\omega_{k}^{2}}\right\}\psi_{2,k} \textup{exp}\left\{-\psi_{2,k} \omega_{k}^{2}\right\}d\omega_{k}^{2} \end{aligned}  $$

Then the hierarchical prior can be represented as

17 $$ {}\begin{aligned}  \boldsymbol{\beta}_{i}|\sigma^{2},\tau_{1}^{2},\cdots,\tau_{2p_{i}}^{2},\omega_{1}^{2},\cdots,\omega_{p_{i}}^{2} &\sim \textup{N}_{p}(\mathbf{0},\sigma^{2}\Sigma_{\beta}),\\ \tau_{j}^{2}|\psi_{1,j} &\sim \textup{Exp}(\psi_{1}), j=1,2,\cdots,2p_{i} \\ \omega_{k}^{2}|\psi_{2,k} &\sim \textup{Exp}(\psi_{2}), k=1,2,\cdots,p_{i}. \end{aligned}  $$

The hyper parameters, *ψ*_1,*j*_ and *ψ*_2,*k*_, are equivalent to the tuning parameters that adjust the sparsity of ***β***_*i*_ and *Δ****β***_*i*_. We consider the class of Gamma prior on them, namely Gamma(a,b), where *a* and *b* can be pre-specified appropriate values so that the hyper priors for *ψ*_1,*j*_ and *ψ*_2,*k*_ are essentially noninformative. It should be noted that here we employ adaptive tuning parameters for each penalized term in line with the adaptive Lasso [33] to improve the accuracy and robustness of estimation.

From Eq. (17), we see that $\boldsymbol {\beta }_{i}|\sigma ^{2},\tau _{1}^{2},\cdots,\tau _{2p_{i}}^{2},\omega _{1}^{2},\cdots,\omega _{p_{i}}^{2}$ is in line with multivariate normal distribution, according to Eq. (16), it is deduced from

18 $$ {}\begin{aligned}  &\pi\left(\boldsymbol{\beta}_{i}|\sigma^{2},\tau_{1}^{2},\cdots,\tau_{2p_{i}}^{2},\omega_{1}^{2},\cdots,\omega_{p_{i}}^{2}\right) \\=& \prod_{j=1}^{2p_{i}} \textup{N}\left(\beta_{i,j}|0,\sigma^{2}\tau_{j}^{2}\right)\prod_{k=1}^{p_{i}} \textup{N}\left(\beta_{i,p_{i}+k}-\beta_{i,k}|0,\sigma^{2}\omega_{k}^{2}\right) \\& \propto \textup{exp}\left\{ -\frac{1}{2\sigma^{2}} \left(\sum_{j=1}^{2p_{i}}\frac{\beta_{i,j}^{2}}{\tau_{j}^{2}}+\sum_{k=1}^{p_{i}}\frac{\left(\beta_{i,p_{i}+k}-\beta_{i,k}\right)^{2}}{\omega_{k}^{2}} \right)\right\}, \end{aligned}  $$

where

19 $$ {}\begin{aligned}  &\sum_{j=1}^{2p_{i}}\frac{\beta_{i,j}^{2}}{\tau_{j}^{2}}+\sum_{k=1}^{p_{i}}\frac{\left(\beta_{i,p_{i}+k}-\beta_{i,k}\right)^{2}}{\omega_{k}^{2}} = \beta_{i,1}^{2}\left(\frac{1}{\tau_{1}^{2}}+\frac{1}{\omega_{1}^{2}}\right)\\&+\cdots+\beta_{i,p_{i}}^{2}\left(\frac{1}{\tau_{p_{i}}^{2}}+\frac{1}{\omega_{p_{i}}^{2}}\right) + \beta_{i,p_{i}+1}^{2}\left(\frac{1}{\tau_{p_{i}+1}^{2}}+\frac{1}{\omega_{1}^{2}}\right)+\cdots\\&+\beta_{i,2p_{i}}^{2}\left(\frac{1}{\tau_{2p_{i}}^{2}}+\frac{1}{\omega_{p_{i}}^{2}}\right) -2\sum_{k=1}^{p_{i}}\frac{\beta_{i,p_{i}+k}\beta_{i,k}}{\omega_{k}^{2}}. \end{aligned}  $$

Therefore, $\boldsymbol {\beta }_{i}|\sigma ^{2},\tau _{1}^{2},\cdots,\tau _{2p_{i}}^{2},\omega _{1}^{2},\cdots,\omega _{p_{i}}^{2}$ is multivariate normal distributed with mean vector **0** and covariance matrix $\sigma ^{2}\Sigma _{\boldsymbol {\beta }_{i}}$ with

20 $$ {}\begin{aligned}  \Sigma_{\boldsymbol{\beta}_{i}}^{-1}= \left[ \begin{array}{cccccc} \frac{1}{\tau_{1}^{2}}+ \frac{1}{\omega_{1}^{2}}& \cdots & 0 & -\frac{1}{\omega_{1}^{2}} & \cdots & 0\\ \vdots & \ddots & \vdots & \vdots & & \vdots\\ 0 & \cdots & \frac{1}{\tau_{p_{i}}^{2}}+ \frac{1}{\omega_{p_{i}}^{2}} & 0 & \cdots & -\frac{1}{\omega_{p_{i}}^{2}}\\ -\frac{1}{\omega_{1}^{2}} & \cdots & 0 & \frac{1}{\tau_{p_{i}+1}^{2}}+ \frac{1}{\omega_{1}^{2}} & \cdots & 0\\ \vdots & \ddots & \vdots & \vdots & \ddots & \vdots\\ 0 & \cdots & -\frac{1}{\omega_{p_{i}}^{2}} & 0 & \cdots & \frac{1}{\tau_{2p_{i}}^{2}}+ \frac{1}{\omega_{p_{i}}^{2}} \end{array}.\right] \end{aligned}  $$

The hierarchical prior in Eqs. (16) and (17) implement the optimization problem as described in Eq. (15). We assign *σ*^2^ an Inverse-Gamma prior with hyper parameters *ν*_0_/2 and *η*_0_/2, the hyper parameters can be pre-specified appropriate values. With the likelihood

21 $$ \begin{aligned}  \mathbf{y}_{i}|\mathbf{W}_{i},\boldsymbol{\beta}_{i},\sigma^{2} \sim \textup{N}_{n}\left(\mathbf{W}_{i}\boldsymbol{\beta}_{i}, \sigma^{2}\mathbf{I}_{n}\right), \end{aligned}  $$

the full conditional posteriors of the hierarchical model can be given by:

22 $$ {}\begin{aligned}  \boldsymbol{\beta}_{i}|\mathbf{y}_{i},\mathbf{W}_{i},\sigma^{2}, \tau_{1}^{2},\cdots,\tau_{2p_{i}}^{2},\omega_{1}^{2},\cdots,\omega_{p_{i}}^{2} &\sim \textup{N}_{n}\left(\mathbf{A}^{-1}\mathbf{W}_{i}^{T}\mathbf{y}_{i}, \sigma^{2}\mathbf{A}^{-1}\right),\\ \mathbf{A}&=\mathbf{W}_{i}^{T}\mathbf{W}_{i}+\Sigma_{\boldsymbol{\beta}_{i}}^{-1}\\ \sigma^{2}|\mathbf{y}_{i},\mathbf{W}_{i}, \boldsymbol{\beta}_{i}, \tau_{1}^{2},\cdots, \tau_{2p_{i}}^{2},\omega_{1}^{2},\cdots,\omega_{p_{i}}^{2} &\sim \textup{IGamma}\left(\frac{\nu}{2},\frac{\eta}{2}\right), \\ \nu&=n+2p_{i}+\nu_{0}-1,\\ \eta&=\left\| \mathbf{y}_{i}\! -\! \mathbf{W}_{i}\boldsymbol{\beta}_{i} \right\|_{2}^{2}\,+\,\boldsymbol{\beta}_{i}^{T}\Sigma_{\boldsymbol{\beta}_{i}}^{-1}\boldsymbol{\beta}_{i}\!+\eta_{0}\\ \frac{1}{\tau_{j}^{2}}|\boldsymbol{\beta}_{i,j}, \sigma^{2}, \psi_{1,j} &\sim \textup{IGauss}\left(\mu_{1}, \lambda_{1}\right), j\!=1,\cdots,2p_{i} \\ \mu_{1}&=\sqrt{\frac{2\psi_{1,j}\sigma^{2}}{\beta_{i,j}^{2}}},\lambda_{1}=2\psi_{1,j},\\ \frac{1}{\omega_{k}^{2}}|\boldsymbol{\beta}_{i,k},\boldsymbol{\beta}_{i,p_{i}+k}, \sigma^{2}, \psi_{2,k} &\sim \textup{IGauss}(\mu_{2}, \lambda_{2}), k=1,\cdots,p_{i} \\ \mu_{2}&=\sqrt{\frac{2\psi_{2,k}\sigma^{2}}{\left(\beta_{i,p_{i}+k}-\beta_{i,k}\right)^{2}}},\lambda_{2}\!=2\psi_{2,k}\\ \psi_{1,j}|\tau_{1}^{2}, \cdots, \tau_{2p_{i}}^{2},a,b &\sim \textup{Gamma}\left(a+1,b+\tau_{j}^{2}\right), \\j&=1,\cdots,2p_{i}\\ \psi_{2,k}|\omega_{1}^{2}, \cdots, \omega_{p_{i}}^{2},a,b &\sim \textup{Gamma}\left(a+1,b+\omega_{k}^{2}\right), \\k&=1,\cdots,p_{i}. \end{aligned}  $$

Then a Gibbs sampler is used to draw samples iteratively from the above posteriors, and yields posterior estimates of ***β***_***i***_, the uncertainty can also be characterized in a natural way through the credible intervals. The convergence of the Gibbs sampler is monitored by the potential scale reduction factor $\widehat {R}$ as introduced in [37] and the convergence condition is set to $\widehat {R}<1.1$. Once the Gibbs sampler converges, we continue to draw samples for several iterations and average the converged samples of ***β***_*i*_ as the estimations for ***β***_*i*_. Vats [38] and Kyung et al. [34] have proved geometric ergodicity of the Gibbs samplers for the Bayesian fused lasso. Following the conclusion in [38], under the condition of *n*>3, no conditions on *p*_*i*_ are required to fulfil the geometric ergodicity. Thus, the convergence of the Gibbs sampler is expected to be quite speed regardless of the dimension *p*_*i*_.

With the samples for all ***β***_***i***_ drawn from the Gibbs sampler, the posterior mean estimate and corresponding credible interval of $(\mathbf {B}^{(1)}_{i}$, $\mathbf {B}^{(2)}_{i})$ can also be obtained. After applying the Gibbs Sampler on all the *p* models for *i*=1,⋯,*p*, the adjacency matrices of two GRNs **B**^(1)^ and **B**^(2)^ as well as the difference between them *Δ***B** can be easily figured out.

Different from the frequency framework, a Bayesian hierarchical model with penalized prior can shrinkage the regression coefficients but does not produce exactly zero estimates. Several strategies have been proposed to go from a posterior distribution to a sparse point estimate [39–41]. Considering the computing complexity, here we adopt the simplest strategy suggested in [42–44] to preset a threshold value *t*. In the adjacency matrices **B**^(1)^ and **B**^(2)^, only the entries whose absolute value are larger than *t* are retained, all other entries are set to zero. Then the differential GRN can be obtained by computing *Δ***B**=**B**^(1)^−**B**^(2)^. Obviously, there is a trade off between power of detection (PD) and discovery rate (FDR), the smaller *t* is, more edges would be detected in the GRNs, which results in better PD but worse FDR; and reversely, a larger *t* yields worse PD but better FDR. As discussed in [42], the value of the threshold *t* is chosen subjectively. Referring to the threshold value in [42] (*t*=0.1) and [44] (*t*=0.05,0.1,0.2), we set *t*=0.2 for the following computer simulations.

## Results

### Computer Simulations

In this section, we run simulations on synthetic data by applying our proposed BFDSEM algorithm and two state-of-the-art joint differential analysis algorithms: FSSEM and ReDNet, and then compare the performance in terms of PD and FDR for (**B**^(1)^,**B**^(2)^) and *Δ***B**. Since the algorithms may have different performance in DAGs and DCGs, it is commonplace to run simulations on synthetic DAGs and DCGs, respectively.

Following the setup in [13, 20], both DAGs and DCGs under two different conditions are simulated. The simulated data have similar numeric data type and range with corresponding standardized experimental data, so the simulation studies could reflect the performance of the algorithms to some extent. The number of genes *p* varies from 10 to 30 or 50, the sample size *n* varies from 50 to 250. In the following simulations, the number of cis-eQTLs *q* is set as *q*=2*p*, meaning that each gene has two contributing cis-eQTLs. The average number of edges per node *n*_*e*_ which determines the degree of sparsity varies from 1 to 3 or 4.

In detail, an adjacency matrix of a DAG or a DCG **A**^(1)^ is first generated for the GRN under condition 1, then the corresponding adjacency matrix **A**^(2)^ is generated by randomly changing *n*_*d*_ entries of **A**^(1)^, where *n*_*d*_ is approximately equal to 10% of the nonzero entries, and the number of changes from 1 to 0 and from 0 to 1 are equal (denoted by *n*_*c*_). The network matrix of GRN under condition 1 **B**^(1)^ is generated from **A**^(1)^ by replacing its nonzero entries with random values generated from a uniform distribution over $(-1, -0.5)\bigcup (0.5, 1)$. Next, the corresponding network matrix under condition 2 **B**^(2)^ is generated from **A**^(2)^ and **B**^(1)^ by steps as follows: For all $A_{ij}^{(2)}=0$, we set $B_{ij}^{(2)}=0$; for all $A_{ij}^{(2)}=A_{ij}^{(1)}$, we randomly select *n*_*c*_ entries and keep them unchanged, other entries are set as $B_{ij}^{(2)}=B_{ij}^{(1)}$; for all $A_{ij}^{(2)}=1$ but $A_{ij}^{(1)}=0$, we generate $B_{ij}^{(2)}$ from a uniformly distribution over interval $(-1, -0.5)\bigcup (0.5, 1)$. The genotypes of the *q* cis-eQTLs are simulated from an F2 cross. Values 1 and 3 were assigned to two homozygous genotypes, respectively, and value 2 to the heterozygous genotype. Then each entry in **X**^(1)^ and **X**^(2)^ are generated by sampling from {1, 2, 3} with corresponding probabilities {0.25, 0.5, 0.25}. The regulatory effects of corresponding cis-eQTLs are assumed to be 1, so **F**^(1)^ and **F**^(2)^ are simulated by randomly permuting the rows of matrix (**I**_*p*_,**I**_*p*_)^*T*^, where **I**_*p*_ denotes a *p*-dimensional identify matrix. In the following simulations, we assume **F**^(1)^=**F**^(2)^. Each error term in **E**^(1)^ and **E**^(2)^ is independently sampled from a normal distribution with zero mean and variance *σ*^2^. Then, the gene expression matrices **Y**^(1)^ and **Y**^(2)^ can be obtained by computing **Y**^(*k*)^=(**X**^(*k*)^**F**^(*k*)^+**E**^(*k*)^)(**I**−**B**^(*k*)^)^−1^,*k*=1,2.

For each setup of the following simulated networks, 20 replicates are simulated, then the PD and FDR are calculated by averaging the results of all replicates in same setups. The variable selection threshold *t* is defined as 0.2.

**Fig. 1:**
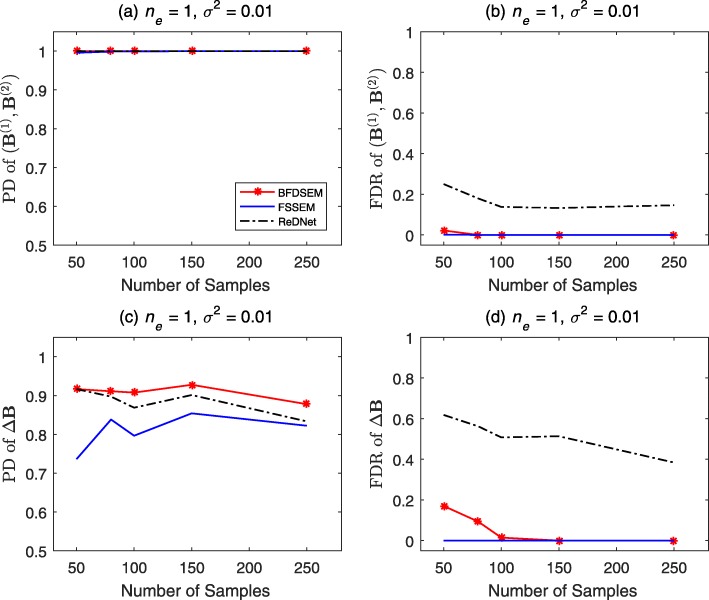
Performance of BFDSEM, FSSEM and ReDNet for DAGs. The number of genes *p*=30, the average number of edges per node *n*_*e*_=1, the noise variance *σ*^2^=0.01, and the sample sizes *n*_1_=*n*_2_ vary from 50 to 250

**Fig. 2:**
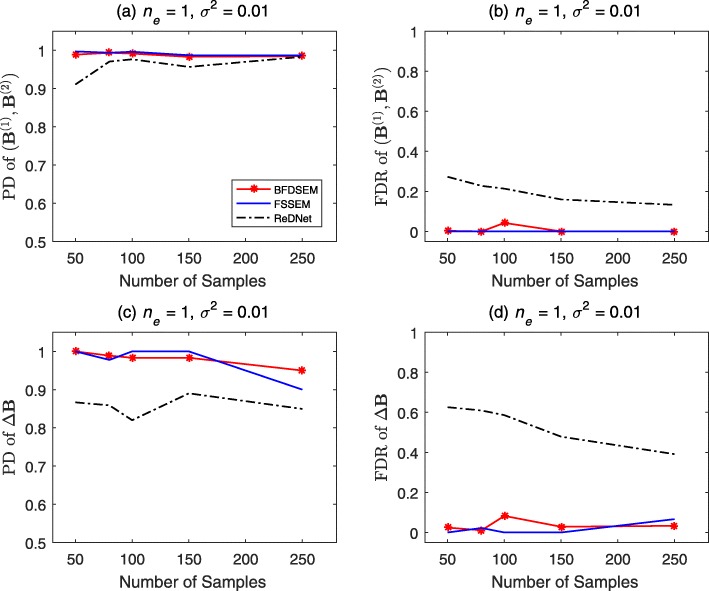
Performance of BFDSEM, FSSEM and ReDNet for DCGs. The number of genes *p*=30, the average number of edges per node *n*_*e*_=1, the noise variance *σ*^2^=0.01, and the sample sizes *n*_1_=*n*_2_ vary from 50 to 250

We depict the results of DAGs and DCGs with *p*=30,*n*_*e*_=1,*σ*^2^=0.01 in Figs. 1 and 2, respectively. First, let us see the results of DAGs in Fig. 1. The PD and FDR of (**B**^(1)^,**B**^(2)^) are shown in Fig. 1a and b. The three algorithms show similar performance in PD, which nearly reaches 1 for all sample sizes. As for the FDR, BFDSEM has similar results with FSSEM, which are better than ReDNet. The PD and FDR of *Δ***B** are depicted in Fig. 1c and d. BFDSEM yields slightly better PD than ReDNet, and more better PD than FSSEM. It offers slightly worse FDR than FSSEM when sample size is ≤100, and much better FDR than ReDNet across all sample sizes. Next to see the results of DCGs in Fig. 2. The PD and FDR of (**B**^(1)^,**B**^(2)^) can be observed in Fig. 2a and b. BFDSEM offers similar or very slightly worse PD and FDR than FSSEM, and provides visual better PD and FDR than ReDNet. The PD and FDR of *Δ***B** are depicted in Fig. 2c and d. BFDSEM and FSSEM perform neck and neck PD and FDR, which are obviously better than ReDNet.

All of the simulation results of DAGs and DCGs under different setups (*n*_*e*_ and *σ*^2^) can be found in Additional files 2, 3, 4, 5: Figure S1-S4. As a whole, BFDSEM generally outperforms ReDNet for all simulation setups. Compared to FSSEM, BFDSEM has similar or slightly better performance for synthetic data sets with *σ*^2^=0.01. When *σ*^2^=0.1, BFDSEM still exhibits similar or better PD for both (**B**^(1)^,**B**^(2)^) and *Δ***B**, but offers worse FDR when sample size is relatively smaller, especially for *Δ***B**.

**Fig. 3:**
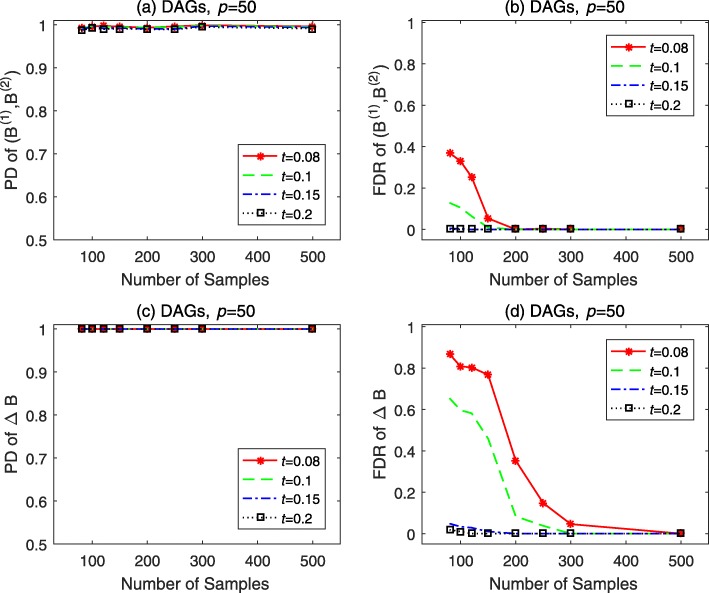
Performance of BFDSEM for DAGs with different Bayesian variable selection threshold *t*. The number of genes *p*=50, the average number of edges per node *n*_*e*_=1, the noise variance *σ*^2^=0.01, the sample sizes *n*_1_=*n*_2_ vary from 80 to 500, and the variable selection threshold *t* ranges in {0.08, 0.1, 0.15, 0.2}

Finally, simulations on DAGs with *p*=50,*n*_*e*_=1,*σ*^2^=0.01 are run to show how does the value of threshold *t* affect the performance of BFDSEM. The simulation results for (**B**^(1)^,**B**^(2)^) and *Δ***B** with *t* ranging in {0.08,0.1,0.15,0.2} and *n* varies from 80 to 500 are depicted in Fig. 3. As shown in Fig. 3a and c, for all values of *t*, the PD of both (**B**^(1)^,**B**^(2)^) and *Δ***B** are similar and all equal to or slightly lower than 1. From Fig. 3b and d, we see that the FDR of (**B**^(1)^,**B**^(2)^) and *Δ***B** still achieve almost perfect results for *t*=0.15 or 0.2. Nevertheless, when *t*=0.08 or 0.1, the FDR of both (**B**^(1)^,**B**^(2)^) and *Δ***B** increase invisibly, especially for *Δ***B** with small sample sizes.

### Real data analysis

We perform differential analysis on a real data set from 42 tumors and their adjacent normal tissues of non-smoking female patients with lung adenocarcinomas. The gene expression levels and genotypes of single nucleotide polymorphisms (SNPs) in this data set were reported in the gene expression omnibus data base GSE33356 by Lu et al. [45]. We preprocessed the raw data in GSE33356 following [20] with R package affy [62] and MatrixEQTL [63], resulting in 1,455 genes with at least one cis-eQTLs at an FDR = 0.01.

To perform more reliable inference, we further selected a smaller subset of the 1,455 genes with the GIANT database. The GIANT database which can be accessed in (http://hb.flatironinstitute.org) contains 144 tissue- and cell lineage-specific GRNs from an integration of data sets covering thousands of experiments contained in more than 14,000 distinct publications. We downloaded the lung network with Top Edges (lung_top.gz) from the GINAT database, the posterior probabilities of each edge can be found in the downloaded network. The edges whose posterior probabilities are less than 0.8 were deleted from the GIANT lung network. Then the 1455 genes with corresponding cis-eQTLs were further filtered with the GIANT lung network, and finally, 15 genes were identified to have interactions with at least one another gene with posterior probability ≥0.80 in the GIANT lung network. The details about these 15 lung genes are described in Additional file 6: Table S1.

Now we can apply BFDSEM on the filtered lung data set containing expression levels of 15 genes and genotypes of corresponding cis-eQTLs under two different conditions (in 42 normal tissues and 42 tumors) to make differential analysis.

**Fig. 4:**
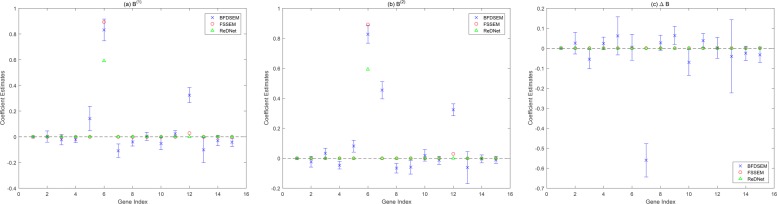
Interval estimate of BFDSEM and point estimates of FSSEM and ReDNet for the first column of **B**^(1)^, **B**^(2)^ and *Δ***B**. Including posterior mean estimates and corresponding 95% equal-tailed credible intervals of BFDSEM, and point estimates of FSSEM and ReDNet for the subset of human lung data set

First, BFDSEM was applied to quantify the uncertainty of the posterior Gibbs sampler by credible intervals. The posterior mean estimates and corresponding 95% equal-tailed credible intervals for **B**^(1)^, **B**^(2)^ and *Δ***B** were estimated and computed, and each result of the first column is depicted in Fig. 4(a)(b)(c), respectively, denoting the regulatory effects of all the 15 genes on the first gene PPP4R2. For comparison, the point estimates of FSSEM and ReDNet are also depicted. Moreover, in Additional files 7, 8, 9: Figure S5-S7 give the results of 100 samples for each estimated edge.

Then we adopt BFDSEM to reconstruct the differential GRN. By directly applying BFDSEM to the original data set with 15 lung genes in 42 tumors and 42 normal tissues, 41 edges were detected. To evaluate the significance of the identified edges, we re-sampled from the original data sets with replacement to obtain 100 bootstraps, each bootstrap also has 42 tumor samples and 42 normal samples. Then BFDSEM is applied to the 100 bootstraps separately, and only the edges that were detected for more than 80 times were retained in the final GRNs. Finally, BFDSEM yielded a GRN with 18 edges for normal lung tissues **B**^(1)^ and a GRN with 17 edges for lung tumors **B**^(2)^. We compared the resulted normal GRN with the GIANT reference network inferred from a large number of samples, and found that 13 of the 18 edges were also in the corresponding GIANT lung network with relatively high confidence, which showed that the GRN inferred by the BFDSEM from only a small number of samples is in accordance with the GIANT lung network in some degree.

**Fig. 5:**
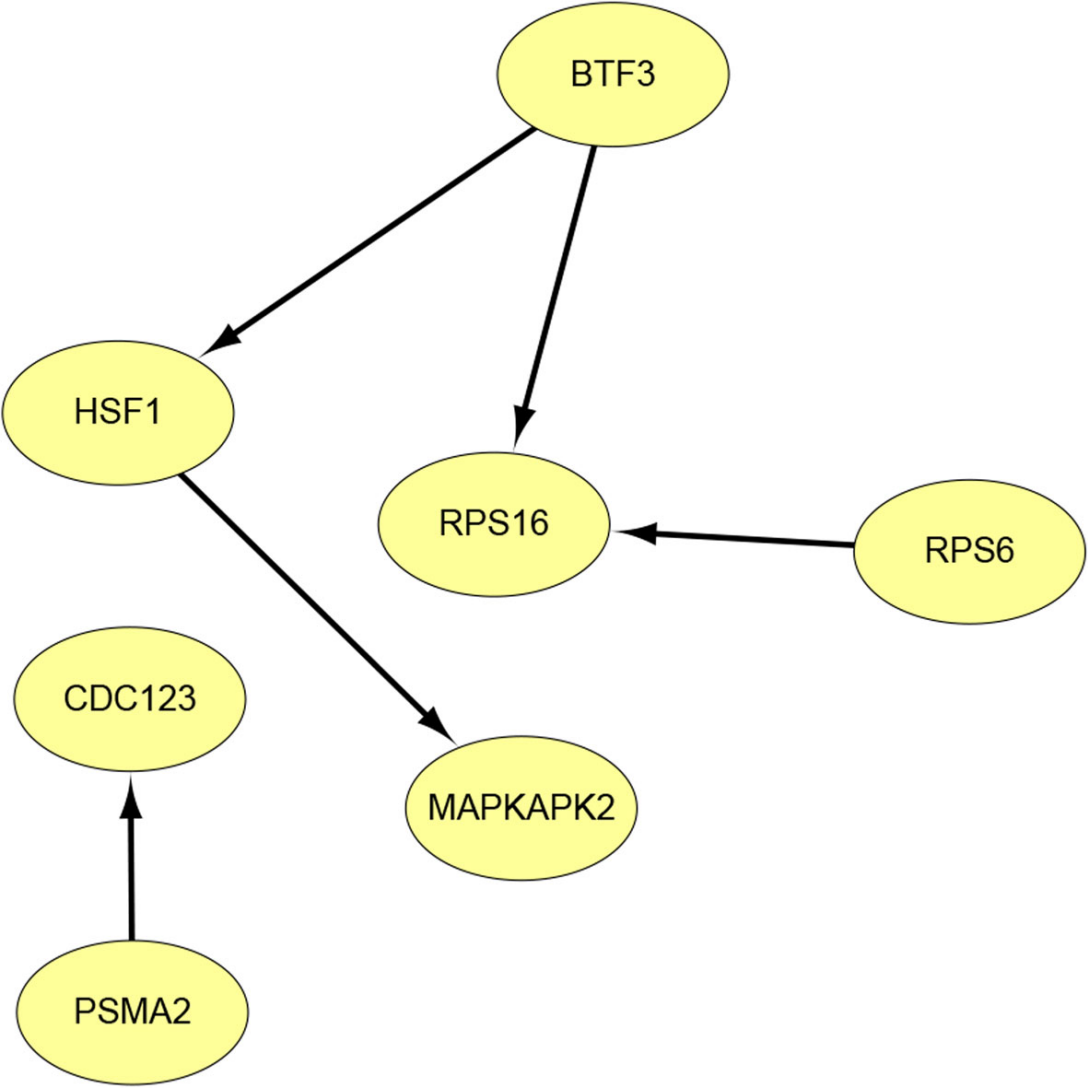
The differential GRN of 15 lung genes identified by the BFDSEM algorithm. Including 7 genes and 5 edges, the other genes that were not involved in the differential GRN were omitted

Since too small changes of the regulatory effects are often of little significance in biological, for a differential GRN identified by *Δ***B**=**B**^(1)^−**B**^(2)^, we only take the entries that satisfy the following condition: $|B_{ij}^{(1)}-B_{ij}^{(2)}|>\textit {min}\left \{\left (B_{ij}^{(1)},B_{ij}^{(2)}\right)\right \}$/5. This criteria was applied to all the 100 bootstraps, and the ultimate differential GRN was obtained by eliminating the edges that were detected for less than 80 times. The identified differential GRN with 7 genes and 5 edges is depicted in Fig. 5, in which the mainly related genes are: BTF3, RPS16, HSF1, RPS6, and MAPKAPK2.

## Discussion

An SEM provides a systematic framework to integrate genetic perturbations with gene expression data to improve inference accuracy, and offers flexibility to model both DAGs and DCGs [13]. FSSEM and ReDNet are two state-of-the-art joint inference algorithms for differential analysis of two similar GRNs modeled with SEMs. The performance of these two joint inference algorithms have been proved much more efficient than naive approaches. The FSSEM algorithm in [20] modeled a penalized negative log-likelihood function and developed a proximal alternative linearize minimization algorithm to infer coefficients. The ReDNet algorithm in [21] re-parameterized the pair-wise SEMs as an integrated model regarding the averaged regulatory effects and differential regulatory effects as coefficients, and then penalized them to realize sparse learning. In this paper, we develop a novel algorithm named BFDSEM for joint inference of two similar GRNs modeled with SEMs. Different from FSSEM and ReDNet, BFDSEM is implemented based on re-parametrization and Bayesian penalized regression with a novel fused prior. First, the original pair-wise SEMs under different conditions are re-parameterized as an integrated linear model that incorporates all related data sources at the first stage; Next, considering the sparsity of the separate GRNs and the differential GRN, a penalized optimization model for the re-parameterized linear model is constructed and a corresponding penalized hierarchical prior is developed; Finally, the full conditional posteriors are deduced and a Gibbs sampler is conducted to draw samples iteratively from the posteriors, then the posterior credible interval and posterior mean estimation can be obtained from the samples.

Compared to FSSEM and ReDNet, the Gibbs sampler in BFDSEM is easy to implement, and not only provides point estimation via the posterior mean or median, but also quantifies the uncertainty via the credible interval automatically. The geometric ergodicity of Gibbs samplers for the Bayesian fused lasso have been proved in Vats [38] and Kyung et al. [34], which means fast convergence of the iterations. In addition, BFDSEM construct the penalized prior directly for the re-parameterized integrated linear model to achieve sparsity of the separate GRNs and differential GRN simultaneously. This approach is much simpler and faster than FSSEM, and can reach similar performance at the same time. ReDNet also re-parameterized the pair-wise SEMs as an integrated model, the adaptive Lasso was applied to achieve sparsity for the averaged GRN and differential GRN, rather than the separate GRNs, which may result in less accurate estimates.

Simulation studies have been run to compare the performance of BFDSEM with FSSEM and ReDNet, the results demonstrated that our BFDSEM algorithm has similar performance with FSSEM, and has better performance than ReDNet. The differential analysis of a real data set with 15 genes of 42 lung tumors and 42 normal tissues has been made to infer the underlying GRNs and differential GRN. The resulted normal GRN was demonstrated in good agreement with the GIANT reference network and the identified differential GRN contained 5 highly related genes. The 5 genes have been demonstrated to be related to lung cancer and some other kinds of cancers by experimental approaches in previous literatures. Specifically, BTF3 was confirmed aberrantly in various cancer tissues such as gastric cancer tissues [47, 48], prostate cancer tissues [49], colorectal cancer tissues[50] and pancreatic cancer cells [51]; RPS16 was found dysregulated in disc degeneration, which is one of the main causes of low back pain [52]; HSF1 influenced the expression of heat shock proteins as well as other activities like the induction of tumor suppressor genes, signal transduction pathway, and glucose metabolism. Its associations with gastric cancer [53], breast cancer and two of the studied SNPs correlated significantly with cancer development [54] have been proved; RPS6 was declared closely relevant to the non-small cell lung cancer (NSCLC) [55], the renal cell carcinoma [56] and some other cancers [57, 58]; MAPKAPK2 was demonstrated to contribute to tumor progression by promoting M2 macrophage polarization and tumor angiogenesis [59].

There are still some limitations of the BFDSEM algorithm: First, the selection of the Bayesian variable threshold *t* is somewhat arbitrary to some extent, an improper *t* may lead to less accurate results; Next, despite the apparent theoretical safeguard of geometric ergodicity, when *p*/*n* is large enough, it may be possible for the Gibbs samplers to converge at a slower rate [38, 60], thereby the uncertainty quantification may also be compromised; Moreover, the proposed re-parametrization method only supports pair-wise data sets with the same sample size. A natural direction for future research would be to investigate solutions for these limitations.

## Conclusion

The differential analysis of pair-wise GRNs under different conditions is as important as the inference of single GRNs. In this paper, we develop a novel Bayesian fused differential analysis algorithm for GRNs modeled with SEMs, named BFDSEM, which provides valuable tool for joint inference of GRNs under two different conditions. To our knowledge, our BFDSEM algorithm is the first Bayesian inference method for joint analysis of GRNs modeled with SEMs.

## Availability and Requirements

**Project name:** BFDSEM.**Project home page:** Not applicable.**Operating system(s):** Platform independent.**Programming language:** Matlab.**Other requirements:** None.**License:** None.**Any restrictions to use by non-academics:** None.

## Supplementary information

**Additional file 1** Software. Software package that implements the BFDSEM algorithm.

**Additional file 2** Figure A1. Performance of BFDSEM, FSSEM and ReDNet for DAGs with different setups. The number of genes *p*=10, the average number of edges per node *n*_*e*_=1,3 or 4, the noise variance *σ*^2^=0.01 or 0.1, and the sample sizes *n*_1_=*n*_2_ vary from 50 to 250.

**Additional file 3** Figure A2. Performance of BFDSEM, FSSEM and ReDNet for DAGs with different setups. The number of genes *p*=30, the average number of edges per node *n*_*e*_=1,3 or 4, the noise variance *σ*^2^=0.01 or 0.1, and the sample sizes *n*_1_=*n*_2_ vary from 50 to 250.

**Additional file 4** Figure A3. Performance of BFDSEM, FSSEM and ReDNet for DCGs with different setups. The number of genes *p*=10, the average number of edges per node *n*_*e*_=1,3 or 4, the noise variance *σ*^2^=0.01 or 0.1, and the sample sizes *n*_1_=*n*_2_ vary from 50 to 250.

**Additional file 5** Figure A4. Performance of BFDSEM, FSSEM and ReDNet for DCGs with different setups. The number of genes *p*=30, the average number of edges per node *n*_*e*_=1,3 or 4, the noise variance *σ*^2^=0.01 or 0.1, and the sample sizes *n*_1_=*n*_2_ vary from 50 to 250.

**Additional file 6** Table A1. A table including the Entrez ID, gene names, corresponding eQTL ID, aliases and brief description of the 15 filtered lung genes.

**Additional file 7** Figure A5. The coefficient estimates of BFDSEM, FSSEM and ReDNet for the normal GRN of human lung. Depict the estimate of all the 225 edges in the normal GRN, including 100 samples for each edge drawn from the Gibbs sampler of BFDSEM (×), and point estimates of FSSEM (∘) and ReDNet (△).

**Additional file 8** Figure A6. The coefficient estimates of BFDSEM, FSSEM and ReDNet for the tumor GRN of human lung. Depict the estimate of all the 225 edges in tumor GRN, including 100 samples for each edge drawn from the Gibbs sampler of BFDSEM (×), and point estimates of FSSEM (∘) and ReDNet (△).

**Additional file 9** Figure A7. The coefficient estimates of BFDSEM, FSSEM and ReDNet for the differential GRN of human lung. Depict the estimate of all the 225 edges in the differential GRN, including 100 samples for each edge drawn from the Gibbs sampler of BFDSEM (×), and point estimates of FSSEM (∘) and ReDNet (△).

## Data Availability

The source code and the human lung data set used to make differential analysis are freely available upon request.

## Abbreviations

CNVs: Copy number variants
DAG: Direct acyclic
DCG: Direct cyclic
eQTL: Expression quantitative trait loci
FDR: False discovery rate
GRN: Gene regulatory network
GRN; GRN; PD: Power of detection
SEM: Structural equation model
