# Supplementary figures and images for: Bayesian differential analysis of gene regulatory networks exploiting genetic perturbations
Source: BMC Bioinformatics. 2020 Jan 9;21:12. doi: 10.1186/s12859-019-3314-3 (PMC6953167; doi:10.1186/s12859-019-3314-3)

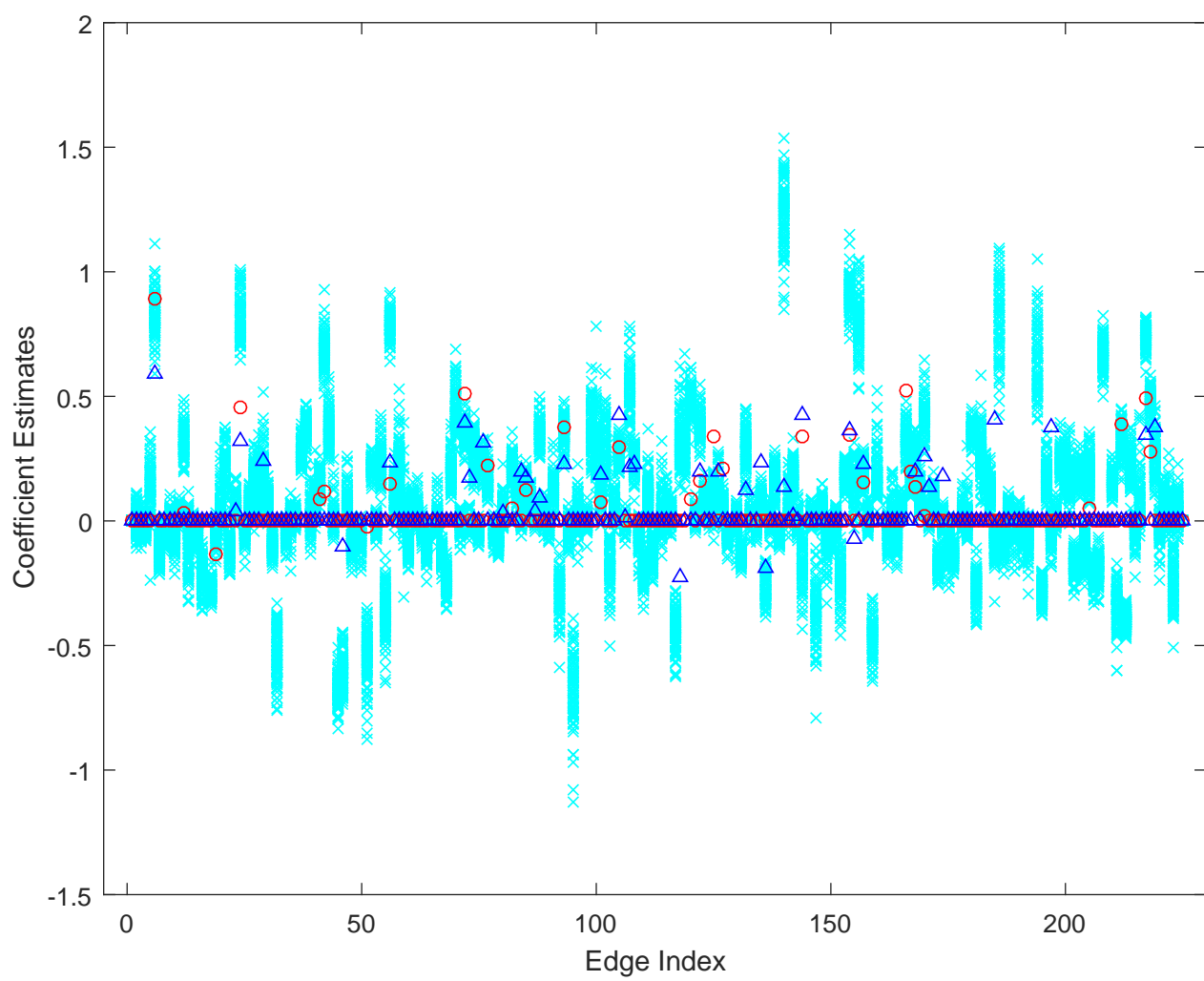

Supplement: Supplementary file 7 — Additional file 7 Figure A5. The coefficient estimates of BFDSEM, FSSEM and ReDNet for the normal GRN of human lung. Depict the estimate of all the 225 edges in the normal GRN, including 100 samples for each edge drawn from the Gibbs sampler of BFDSEM (×), and point estimates of FSSEM (∘) and ReDNet (△). [file 12859_2019_3314_MOESM7_ESM.pdf]

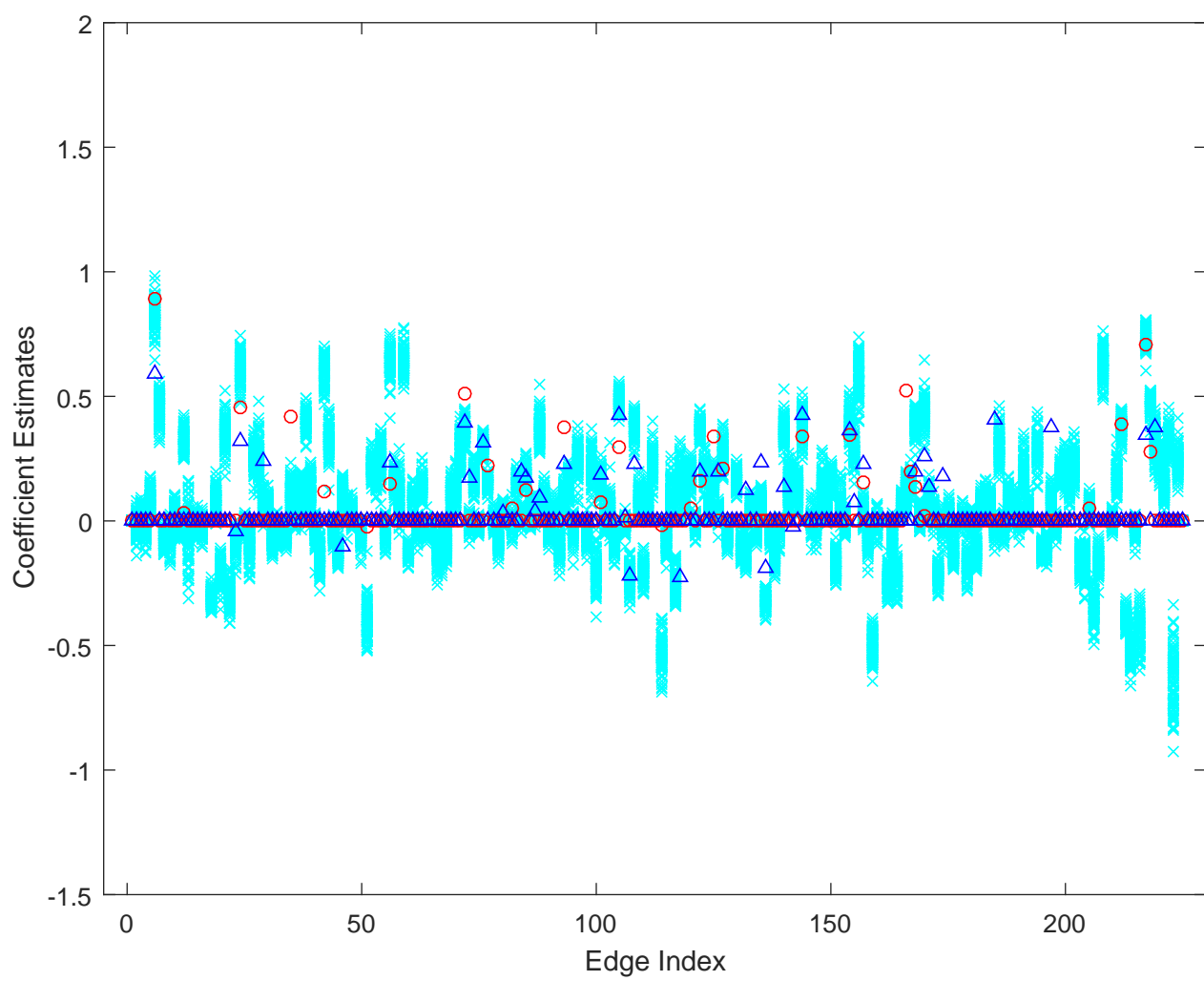

Supplement: Supplementary file 8 — Additional file 8 Figure A6. The coefficient estimates of BFDSEM, FSSEM and ReDNet for the tumor GRN of human lung. Depict the estimate of all the 225 edges in tumor GRN, including 100 samples for each edge drawn from the Gibbs sampler of BFDSEM (×), and point estimates of FSSEM (∘) and ReDNet (△). [file 12859_2019_3314_MOESM8_ESM.pdf]

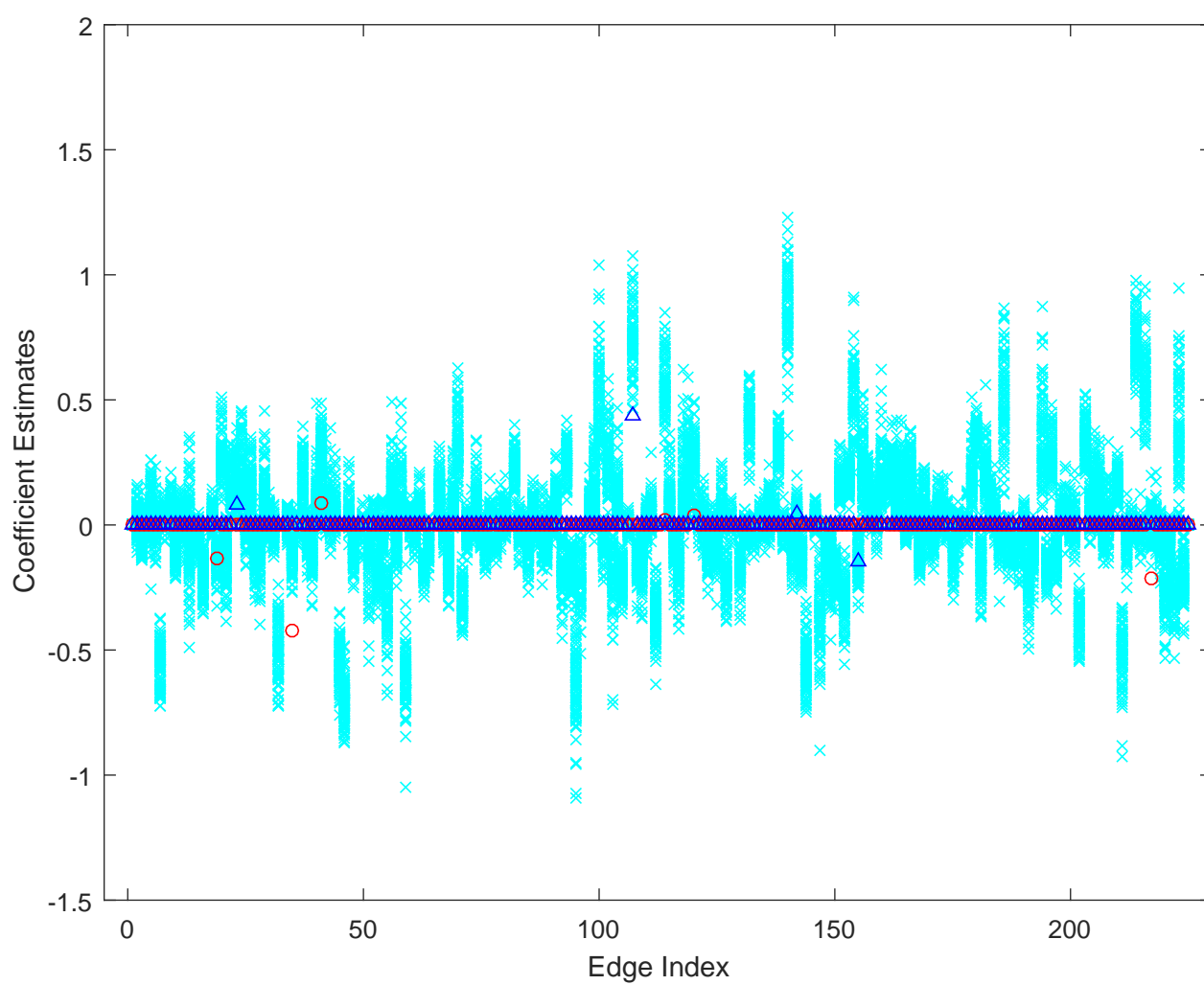

Supplement: Supplementary file 9 — Additional file 9 Figure A7. The coefficient estimates of BFDSEM, FSSEM and ReDNet for the differential GRN of human lung. Depict the estimate of all the 225 edges in the differential GRN, including 100 samples for each edge drawn from the Gibbs sampler of BFDSEM (×), and point estimates of FSSEM (∘) and ReDNet (△). [file 12859_2019_3314_MOESM9_ESM.pdf]
